# Supplementary material for: Enteric nervous system regeneration and functional cure of experimental digestive Chagas disease with trypanocidal chemotherapy
Source: Nat Commun. 2024 May 23;15:4400. doi: 10.1038/s41467-024-48749-5 (PMC11116530; doi:10.1038/s41467-024-48749-5)
Supplement: Supplementary file 1 — Supplementary Information [file 41467_2024_48749_MOESM1_ESM.pdf]

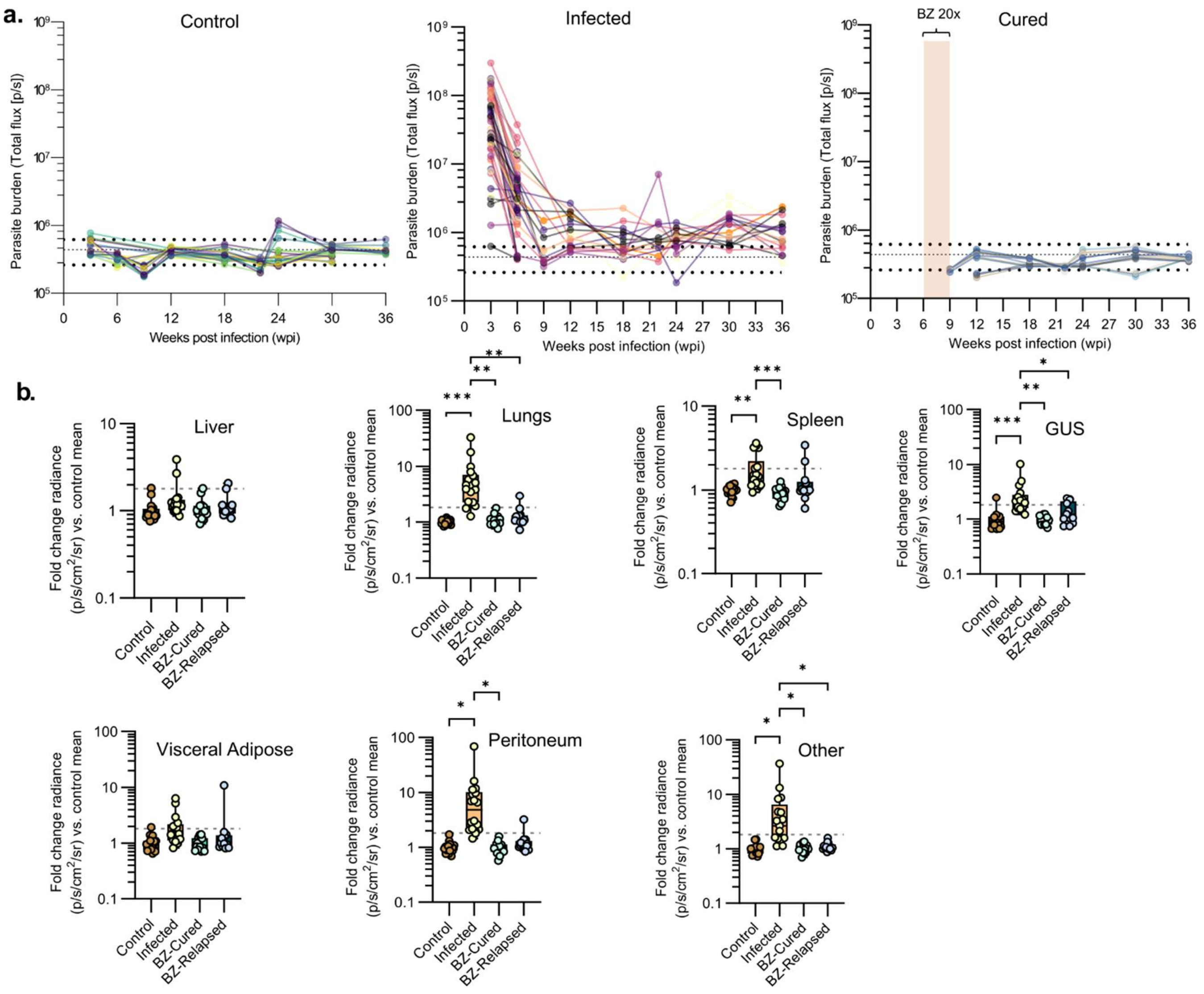

Supplementary Figure 1: Additional *in vivo* and *ex vivo* bioluminescence analysis after benznidazole treatment initiated at 6 weeks post-infection. a, Line plots show *in vivo* parasite bioluminescence signals as total flux of individual C3H/HeN control (*n* = 20, except *n* =15 at 3 and 12 wpi, *n* = 13 at 6 wpi, *n* = 10 at 9, 22 and 36 wpi), infected with TcI-JR (infected; *n* = 18, except *n* = 50 at 3 wpi, *n* = 47 at 6 wpi *n* = 10 at 9 and 22 wpi) and treated with benznidazole (BZ-Cured; *n* = 14, except *n* = 7 at 9 wpi, *n* = 15 at 12 and 18 wpi, and *n* = 6 at 22 wpi) C3H/HeN mice over time. Horizontal lines show thresholds as the average auto-luminescence detected in uninfected controls (dashed) ± 2SD (dotted). Dashed lines show threshold as limit of bioluminescence detection ± 2SD. **b**, Box plots show parasite loads (*ex vivo* bioluminescence) in the liver, lungs, spleen, GUS, visceral adipose, peritoneum and carcass (other) of control (*n* = 19; except GUS, peritoneum and other *n* = 16), infected (*n* = 18; except GUS and other *n* = 17), BZ-Cured (*n* = 14) and BZ-Relapsed (*n* = 13) mice. Data expressed as mean fold change in bioluminescent radiance vs. uninfected control mean. Dashed threshold line is the mean +2SD for an internal control, i.e. (empty) region of interest. All box plot data are expressed as median with minimum and maximum values of each dataset as whiskers and bounds of box as interquartile range. Statistical significance was tested using one-way ANOVA followed by Tukey's HSD test (Only significant differences are annotated: *$P < 0.05$, **$P < 0.01$, *** $P < 0.001$).

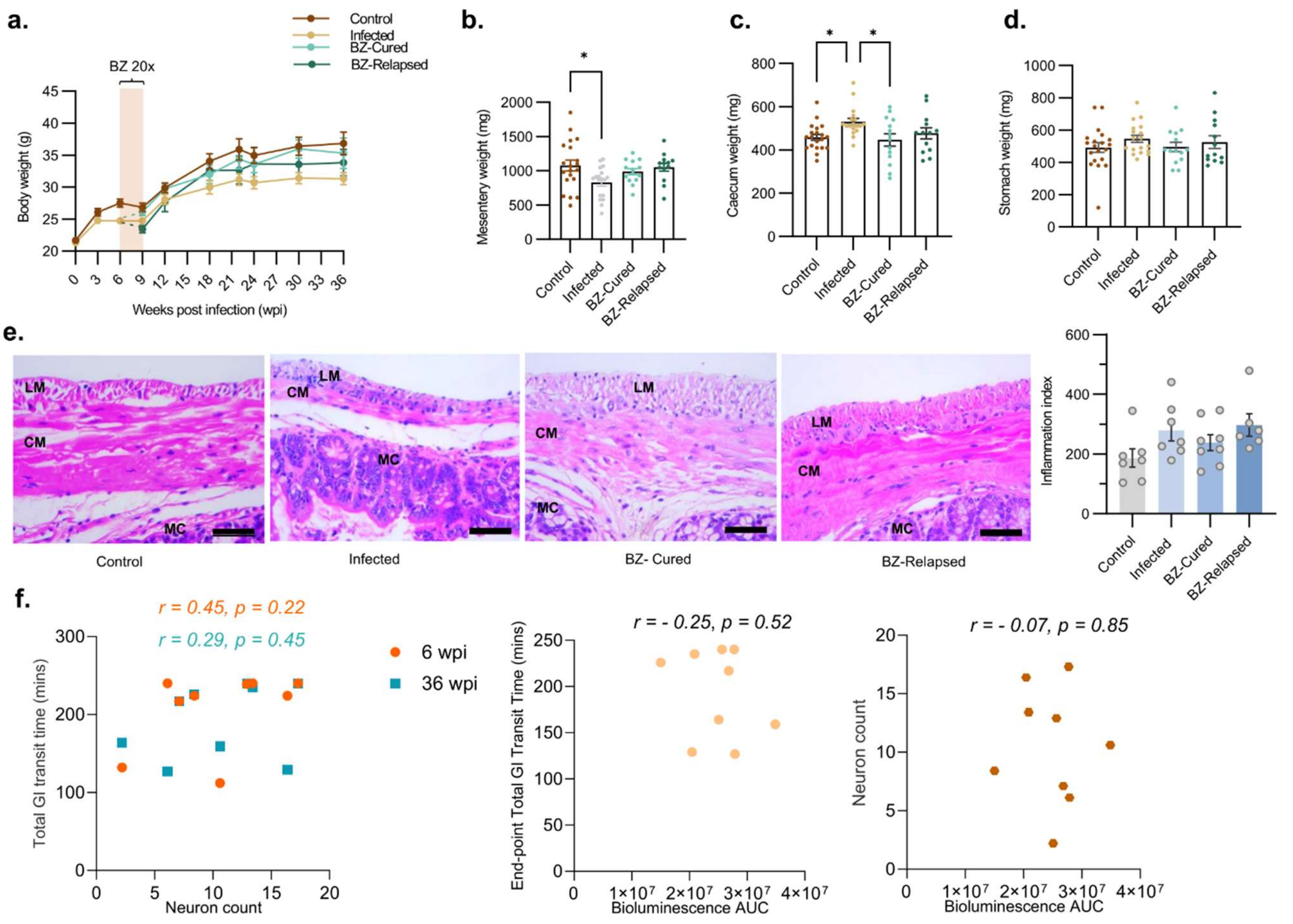

Supplementary Figure 2: Additional GI assays after benznidazole treatment initiated at 6 weeks post-infection. a, Body weight line plots of uninfected control (*n* = 15; except *n* = 10 at 3, 6, 9, 22 and 36 wpi), infected untreated (*n* = 10, except *n* = 25 at 0 and 3 wpi, and *n* = 24 at 6 wpi), benznidazole treated and cured (BZ-cured; *n* = 6, except *n* = 7 at 9, 12 and 18 wpi) and benznidazole treated and relapsed (BZ-relapsed; *n* = 6) mice. Cream bar on shows the BZ treatment window (6-9 wpi). Bar plots show **b,** mesentery **c,** caecum and **d,** stomach weight of control (*n* = 20), infected (*n* = 18), BZ-Cured (*n* = 14) and BZ-Relapsed (*n* = 13) mice at 36 wpi. **e**, Representative brightfield images of 5 µm thick colon transverse sections stained with haematoxylin-eosin (MC, mucosa; CM circular smooth muscle layer; LM longitudinal smooth muscle layer). Images were taken at 400X magnification, scale bar = 50 µm. All micrographs are representative images of two independent experiments. Adjacent bar plot shows number of nuclei per field to quantify cellular infiltration in control (*n* = 7), infected (*n* = 7), BZ-Cured (*n* = 8) and BZ-Relapsed (*n* = 6) mice. Statistical significance was tested using one-way ANOVA followed by Tukey's HSD test (Only significant differences are annotated: *$P < 0.05$). **f**, Pairwise plots and Pearson correlation statistics for the infected untreated group, showing data points for individual mouse (*n* = 9) GI transit times, colon myenteric plexus neuron counts and cumulative parasite burdens (*in vivo* bioluminescence area under the curve (AUC)).

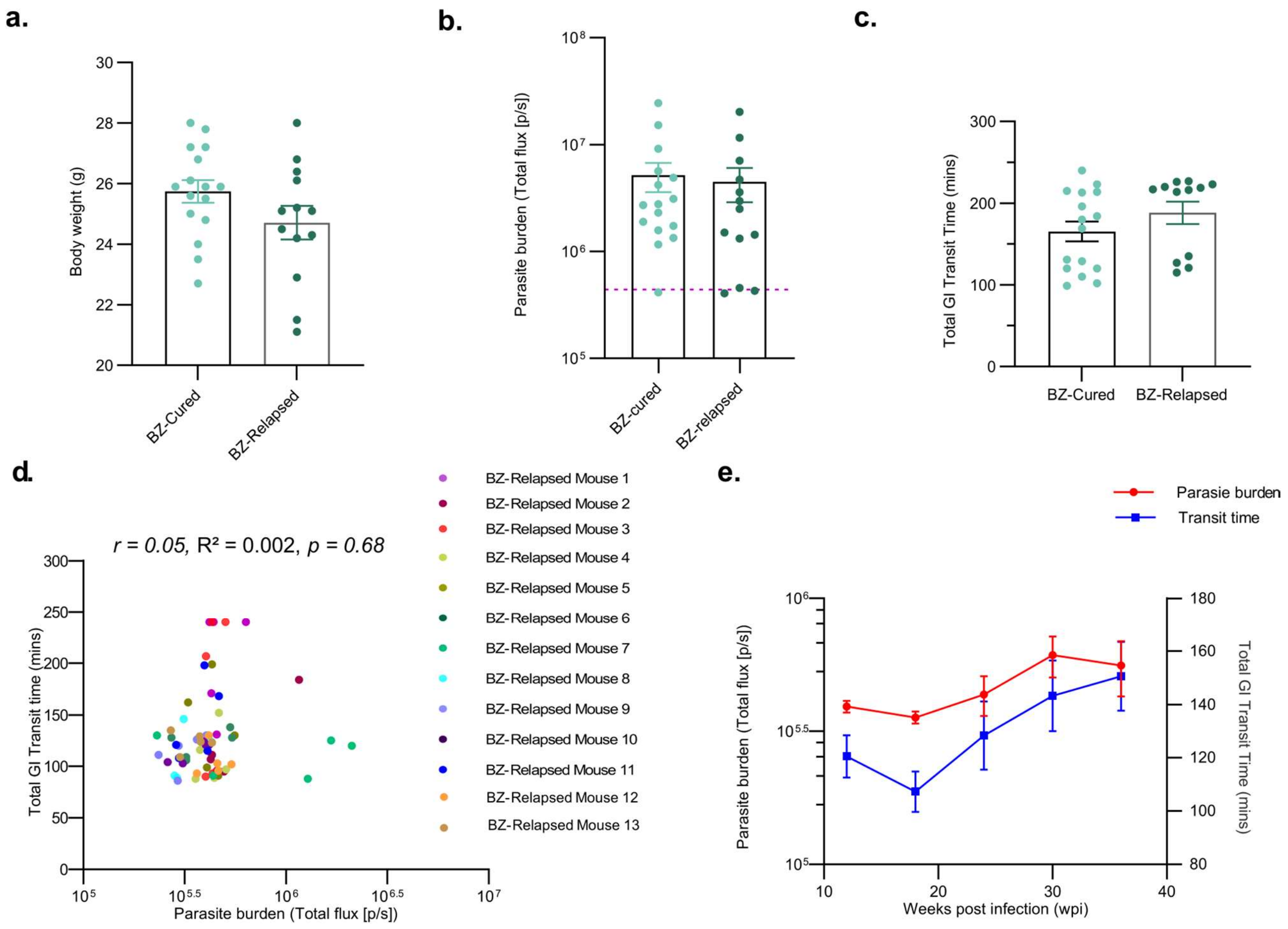

Supplementary Figure 3: Retrospective comparison of cure and relapse groups, and transit times in the context of relapse kinetics. a-c, Bar plots show characteristics of cure and relapse groups at pre-treatment, 6 weeks post-infection: **a,** body weight; **b,** parasite bioluminescence as total flux; and **c,** total GI transit time of BZ-Cured (n = 16) vs BZ-Relapsed (n = 13) mice. Comparison by two-sided t-test showed no significant differences. **d,** Dot plot shows pairwise plot and Pearson correlation statistics for parasite burdens (expressed as total bioluminescent flux) and gastrointestinal transit times for matched time points in individual BZ-Relapsed mice (n = 13 mice, 5 time points per mouse). **e,** Line plots show comparison of mean (+/- SEM) parasite burden (red line) and transit time (blue line) dynamics over time in the same animals.

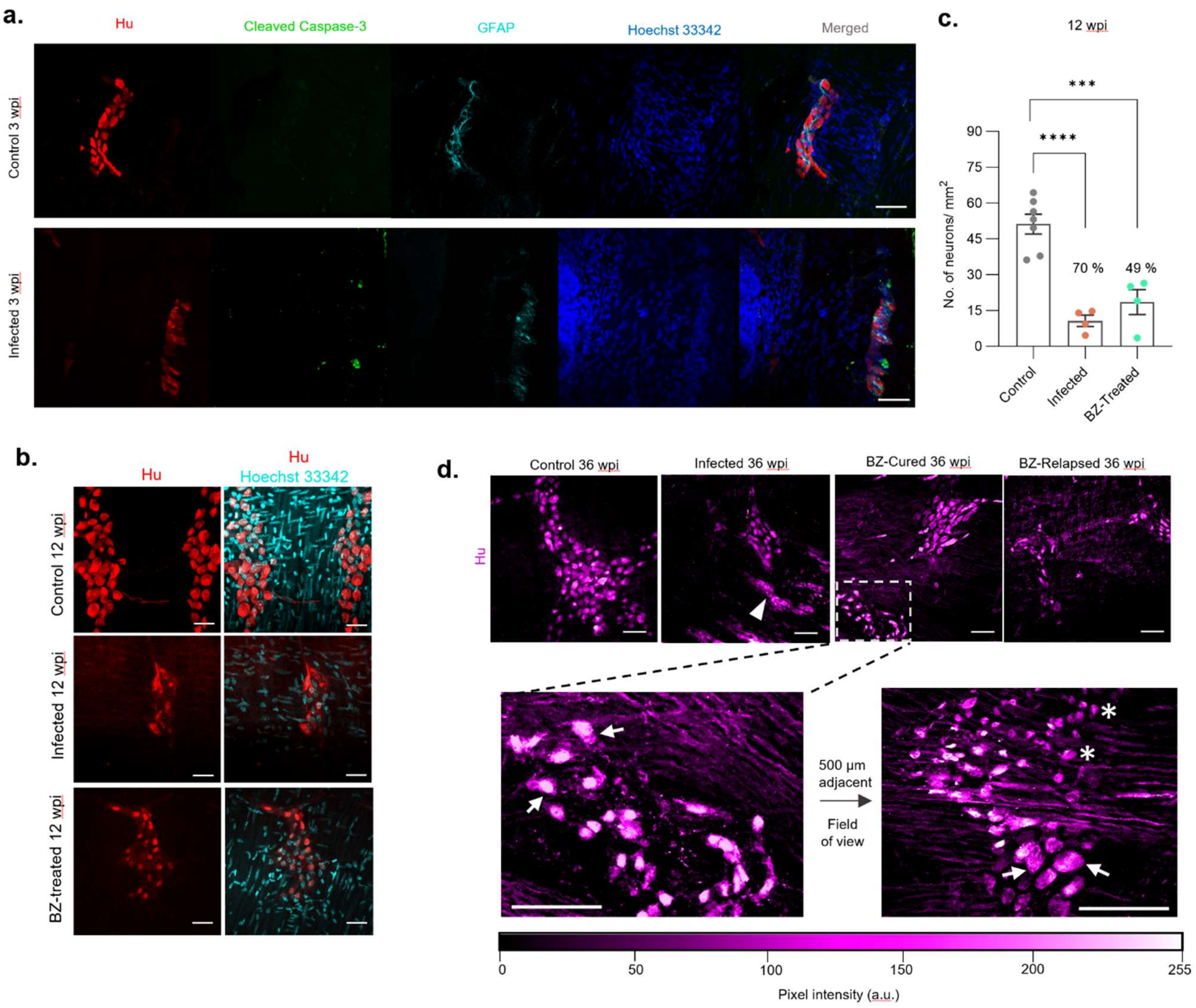

Supplementary Figure 4: Immunofluorescence analysis of enteric neuropathy in *T. cruzi* infections and after benznidazole treatment initiated at 6 weeks. a, Representative confocal images of 3 weeks post-infection (wpi) transverse colon sections from control and TcI-JR infected C3H/HeN mice, immunofluorescently labelled to show acute stage cell loss of Hu^+ (red) neuronal cell bodies (soma) and $GFAP^+$ glial cells (cyan). Cleaved caspase-3 (green) expression shows apoptotic cell death and Hoechst 33342 DNA stain (blue) show cell nuclei. **b**, Representative confocal images of 12 weeks post-infection (wpi; 6 weeks post-treatment, wpt) whole-mount C3H/HeN mouse colon samples from uninfected control (Control), untreated infections with TcI-JR parasites (Infected) and benznidazole treated infections (BZ-treated), immunofluorescently labelled to show changes of Hu^+ soma (red) with and without DNA stain (Hoechst 33342, cyan) in the myenteric plexus. **c,** Bar plot shows quantification of Hu^+ neurons before and after BZ treatment (12 wpi / 6wpt) of control (n = 7), infected (n = 4) and BZ-treated (n = 4) groups. Groups compared using one-way ANOVA, ****$P < 0.0001$. **d**, Representative confocal images of whole-mount colons from control, infected, benznidazole treated cured (BZ-cured) and relapsed (BZ-relapsed) C3H/HeN mice labelled using immunofluorescence to show changes of Hu^+ soma (magenta) in the myenteric plexus at 36 weeks. White arrowhead indicates damaged ganglion (absence of defined Hu^+ soma). Magnified region of BZ-Cured example image shows intact healthy control-like soma, indicated by white arrows. In a 500 µm laterally adjacent region of the same sample, a morphologically heterogeneous neuronal cell population is observed: white arrows indicate intact control-like Hu^+ soma and white stars indicate atypical morphologies. Colour heat map scale shows pixel intensity. Scale bars = 50 µm. All micrographs are representative images of two independent experiments (except (a) is from one independent experiment with 4 technical replicates per group).

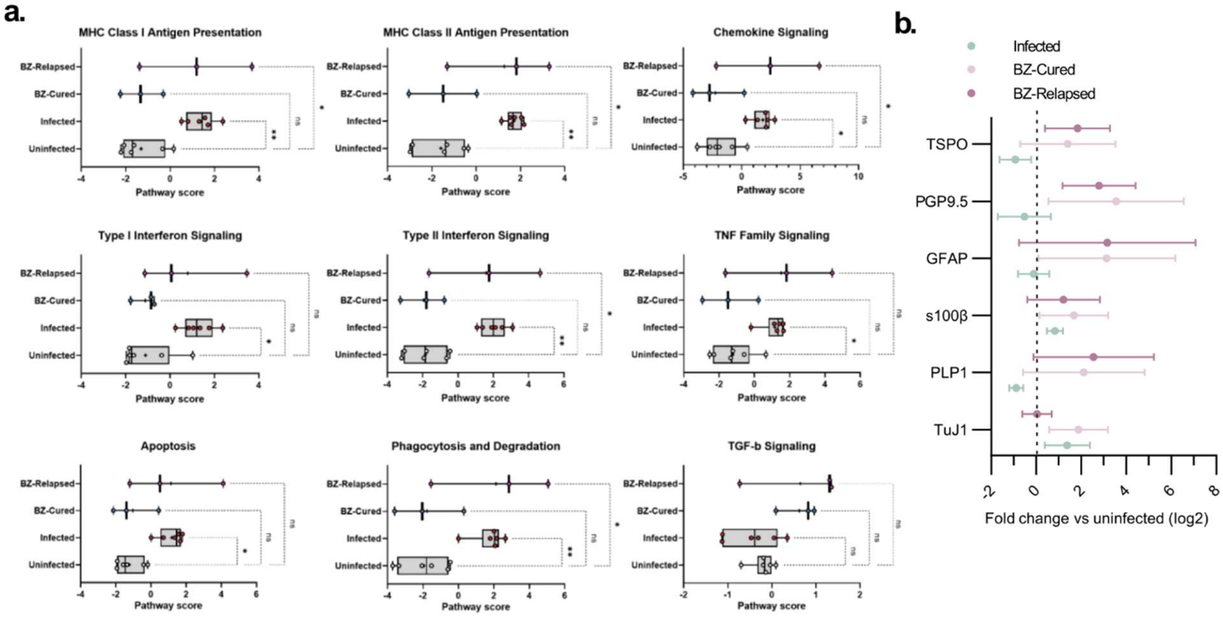

Supplementary Figure 5: Gene expression analysis of immune response and neuro-glial genes. a, Pathway level analysis of differential gene expression patterns in colon tissue at 36 weeks, evaluated using nanostring mouse immunology nCounter codeset. Uninfected control and infected untreated groups n = 6, BZ-Cured and BZ-Relapsed groups, both treated with BZ at 6 weeks, n = 3. Groups compared using one-way ANOVA, $*P < 0.05$, $**P < 0.01$, ns $P > 0.05$. **b,** qPCR analysis of relative expression levels of additional neuronal and glial cell marker genes (n = 4 for all groups).

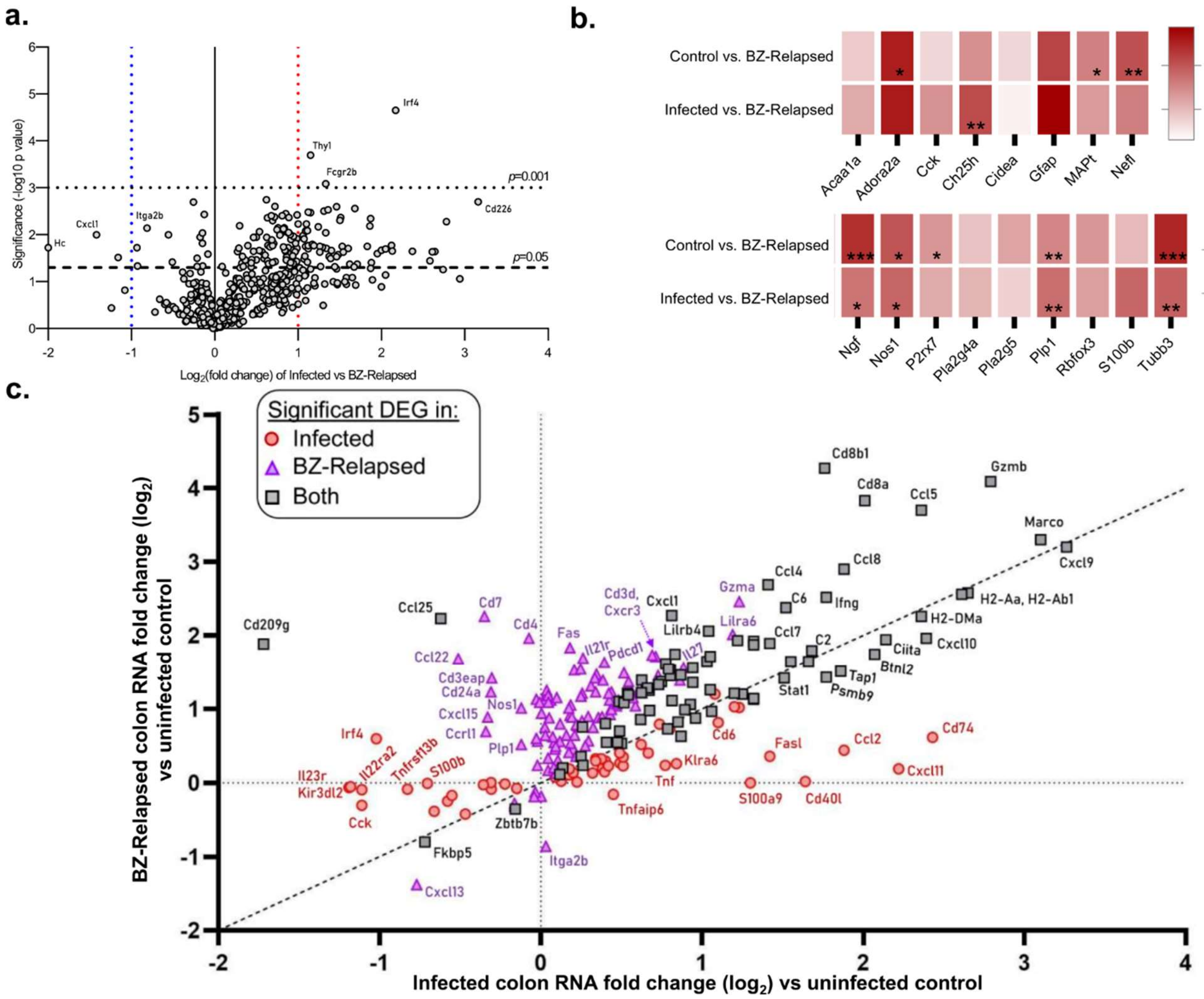

Supplementary Figure 6: Analysis of differential gene expression patterns in post-treatment relapsed infections.

a, Volcano plot of the log2-transformed fold change and significance ($-\log_{10}$ P value) of differentially expressed genes (DEGs) in colon tissue from infected vs. benznidazole treated relapsed (BZ-Relapsed) mice. **b,** Heat maps show relative change in a neuro-glial gene subset between BZ-Relapsed and uninfected control or untreated infected groups. Colour intensity indicates fold change ($\log_2$) expression level. **c,** Comparison of directionality and extent of gene expression change in infected and BZ-Relapsed mice vs controls (n = 230 genes that are significant DEGs in at least one group). Red circles are DEGs specific to the untreated infected group, purple triangles are DEGs specific to the BZ-Relapsed mice and grey squares are DEGs shared by both groups. Diagonal dashed line is the line of equivalence. Vertical and horizontal dashed lines indicate position for genes with identical expression levels as controls in infected and BZ-Relapsed mice respectively. Infected and controls n = 6, BZ-Relapsed n = 3. BZ treatment was at 6 wpi and experimental end-point was at 36 wpi. Statistical significance was determined by 2-tailed, unpaired Student's t-test for each gene (only significant differences are annotated: $*P < 0.05$, $**P < 0.01$, $***P < 0.001$).

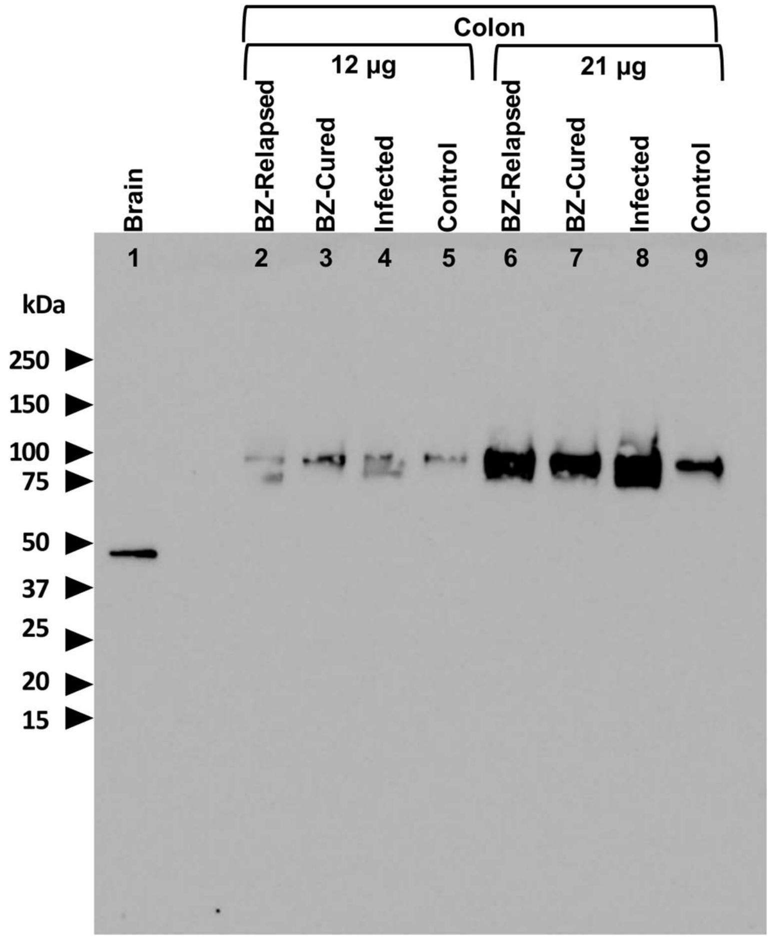

Supplementary Figure 7: Western blot analysis of GFAP in chronic experimental digestive Chagas disease. Representative immunoblot of GFAP protein in colon tissue lysates from uninfected control (Control), TcI-JR infected untreated (Infected), benznidazole treated and cured (BZ-Cured) or relapsed (BZ-Relapsed). Each sample was a pool of n = 3 biological replicates per group. The group and total amount of protein loaded are annotated for wells 2 – 9; well #1 contained a brain tissue sample as a positive control. The blot image is representative of three independent experiments.

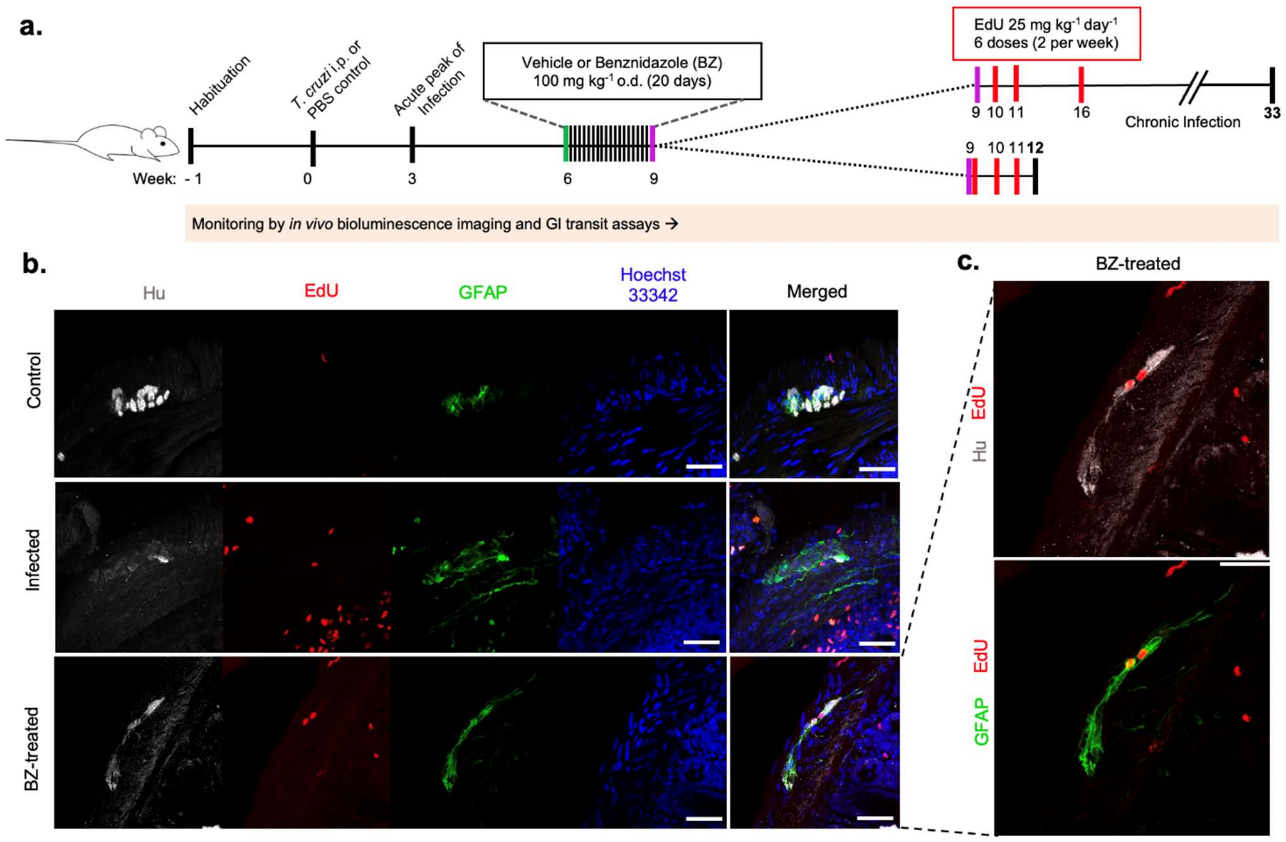

d.

Group	n mice	End-point wpi / wpt	Total EdU⁺ cells observed	Mean EdU⁺ cells observed per mouse	Ganglionic GFAP⁺ Hu⁻ n (%)	Ganglionic GFAP⁻ Hu⁺ n (%)	Ganglionic GFAP⁻ Hu⁻ n (%)	Peri-ganglionic GFAP⁺ Hu⁻ n (%)	Peri-ganglionic GFAP⁻ Hu⁺ n (%)	Peri-ganglionic GFAP⁻ Hu⁻ n (%)
Uninfected Control	6	12 / 6	113	18.8	5 (4.4)	0 (0)	9 (7.9)	7 (6.1)	0 (0)	92 (81.4)
	4	33 / 27	43	10.8	0 (0)	0 (0)	2 (4.6)	35 (81.3)	0 (0)	6 (13.9)
Untreated Infected	5	12 / 6	195	39.0	0 (0)	0 (0)	15 (7.6)	26 (13.3)	0 (0)	154 (78.9)
	4	33 / 27	154	38.5	10 (6.4)	0 (0)	18 (11.6)	103 (66.8)	0 (0)	23 (14.9)
BZ-Treated	4	12 / 6	190	47.5	10 (5.2)	6 (3.1)	34 (17.8)	24 (12.6)	0 (0)	116 (61)
	7	33 / 27	410	58.6	21 (5.1)	3 (0.7)	113 (27.5)	238 (58)	0 (0)	35 (8.5)

Supplementary Figure 8: Post-treatment cellular proliferation analysis by EdU incorporation. a, Schematic representation of the experimental timeline showing mice habituation, course of infection, benznidazole treatment, EdU dosing and follow-up schedules. **b**, Representative immunofluorescence confocal images of 12 wpi transverse colon sections from uninfected control, infected untreated and BZ-treated C3H/HeN mice, labelled to show progeny cells proliferating during EdU pulse(s) (red), Hu^+ neuronal cell bodies (white), $GFAP^+$ glial cells (green) and DNA (Hoechst 33342, blue). **c**, Merged image of BZ-treated sample highlighting co-localisation of EdU with Hu^+ and $GFAP^+$ protein expression. **d**, Table summarising all observations of cells containing EdU and co-localisation with GFAP and Hu protein expression. All micrographs are representative images of one independent experiment with at least four technical replicates per group.

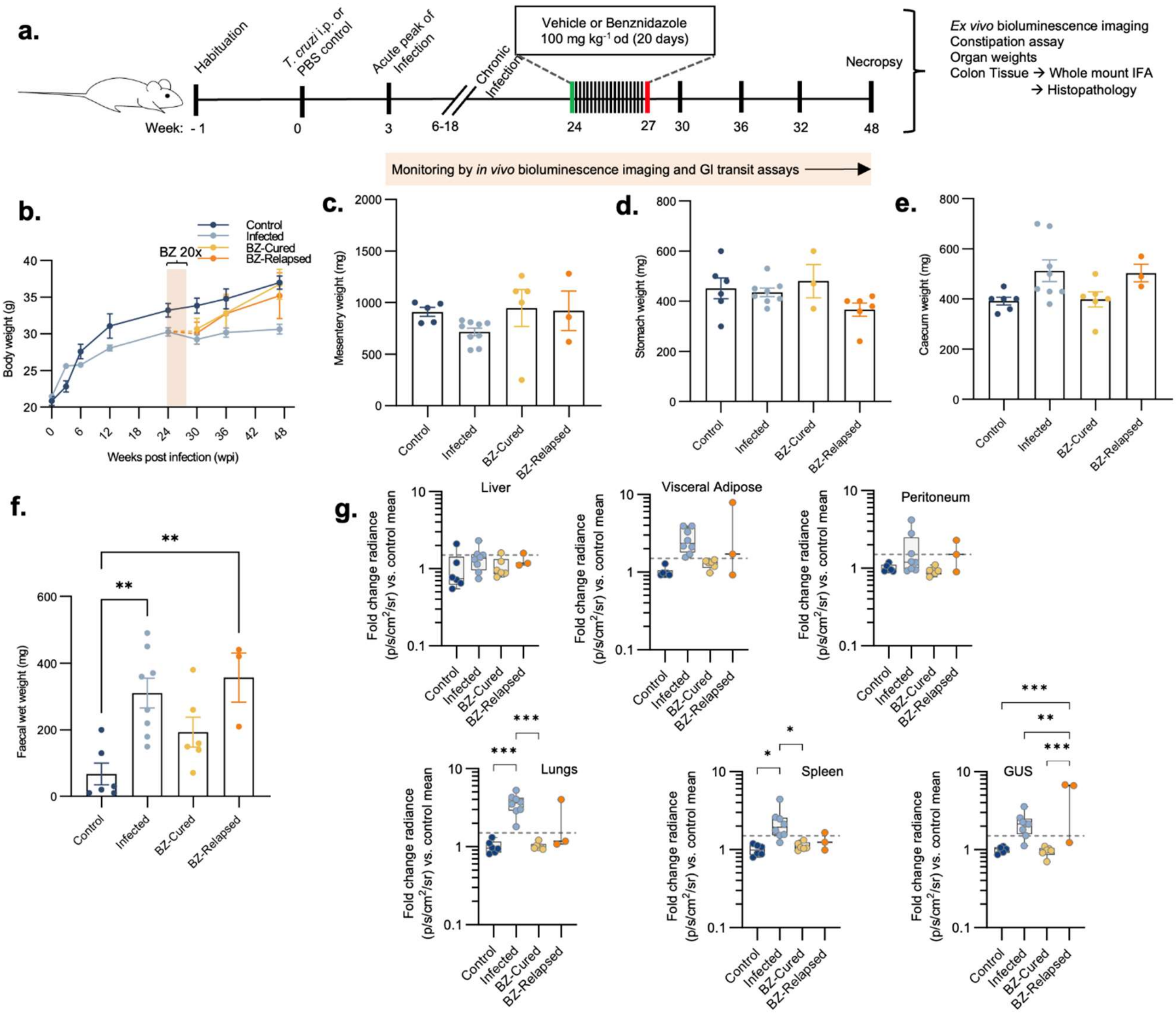

Supplementary Figure 9: Additional GI assays after benznidazole treatment initiated at 24 weeks post-infection. a, Schematic representation of the experimental timeline showing mice habituation, course of infection, treatment and necropsy plan. Groups were female C3H/HeN mice that were uninfected (control), infected with TcI-JR and (i) vehicle-administered (Infected), or treated with benznidazole at 6 wpi and (ii) assessed as parasitologically cured (BZ-Cured), or (iii) assessed as treatment failure (BZ-Relapsed). The experimental end-point was at 48 wpi. **b,** Line plots show body weights; Control n = 5, except n =10 at 0, 3 and 24 wpi; Infected n = 9, except n = 25 at 0 and 3 wpi, n = 20 at 6 wpi, n = 23 at 12 wpi, and n = 22 at 24 wpi; BZ-Cured n = 7, BZ- relapsed; n = 3. Cream bar shows BZ treatment window (24-29 wpi). **c – f,** Bar plots show **c,** mesentery, **d,** stomach **e,** caecum and **f,** faecal wet weight of control (n = 5), infected (n = 8), BZ-Cured (n = 3 (d), 5 (c), or 6 (e,f)) and BZ-Relapsed (n = 3) mice. **g,** Box plots show parasite loads (*ex vivo* bioluminescence) in the liver, visceral adipose, peritoneum, lungs, spleen and genito-urinary system (GUS) of control (n = 6), infected (n = 8), BZ-Cured (n = 6) and BZ-Relapsed (n = 3) mice. Data expressed as mean fold change in bioluminescent radiance vs. uninfected control mean. Dashed threshold line is the mean +2SD for an internal control, i.e. (empty) region of interest. All box plot data are expressed as median with minimum and maximum values of each dataset as whiskers. Statistical significance was tested using one-way ANOVA followed by Tukey's HSD test (Only significant differences are annotated: *$P < 0.05$, **$P < 0.01$, ***$P < 0.001$).

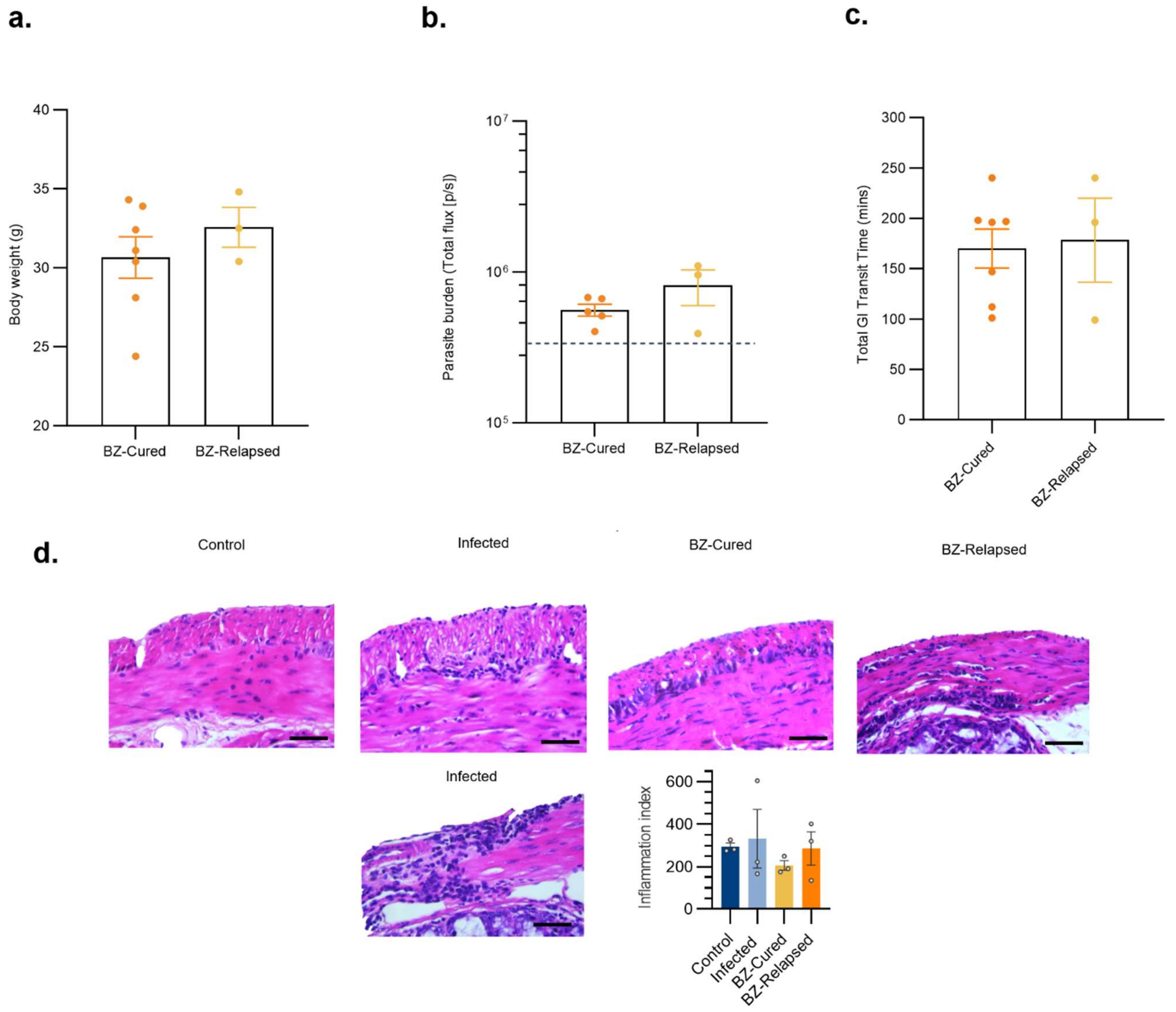

Supplementary Figure 10: Retrospective comparison of cure and relapse group characteristics at point of benznidazole treatment initiation, 24 weeks post-infection. Bar plots show: **a,** body weights; **b,** parasite burdens (bioluminescent total flux); and **c,** total GI transit times of mice that were treated with benznidazole at 24 weeks post-infection and subsequently cured (BZ-Cured; *n* = 7) or not cured (BZ-Relapsed; *n* = 3). **d**, Representative brightfield images of 5 µm thick colon transverse sections stained with haematoxylin-eosin (mucosa bottom; smooth muscle layers top). The second infected image shows the rare, focal nature of cellular infiltration identified during chronic infection. Images were taken at 400X magnification, scale bar = 50 µm. Adjacent bar plot shows number of nuclei per field to quantify cellular infiltration in control, infected, BZ-Cured and BZ-Relapsed C3H/HeN mice (*n* = 3 per group). All micrographs are representative images of two independent experiments.

Supplementary Table 1: Quantitative PCR primer sequences.

**Gene**	**Forward Primer (5'-3')**	**Reverse Primer (5'-3')**	**Amplicon Size (bp)**
Oaz1	GTGGTGGCCTCTACATCGAG	AGCAGATGAAAACGTGGTCAG	120
Tubb3 (TuJ-1)	CAGGGCCATTCTGGTGGACT	TAGTGCCCTTTGGCCCAGTT	135
Gfap	GCAAGAGACAGAGGAGTGGT	CTCTTCCTGTTCGCGCATTT	196
Plp1	GGCGACTACAAGACCACCAT	CAAACTTGTCGGGATGTCCT	154
Tspo	ACTGTATTCAGCCATGGGGTA	ACCATAGCGTCCTCTGTGAAA	75
S100b	GACTCCAGCAGCAAAGGTGAC	CATCTTCGTCCAGCGTCTCCA	228
Uclh1 (PGP9.5)	CCTGTGGTACCATCGGGTTG	GGCTCTATCTTCGGGGGACA	125

Supplementary Data 1: *Ex vivo* bioluminescence values (fold change radiance vs uninfected controls) and post-treatment relapse calls.

This file is provided with the online manuscript.

Supplementary Data 2: Nanostring gene expression data.

The *p* values reported derive from a two-tailed *t*-test on the log-transformed normalized data for each pair of groups under comparison, assuming unequal variance. These are provided as raw p values (Columns headed "P-value") and after adjustment using the Benjamini-Yekutieli procedure (Columns headed "BY.p.value"). This file is provided with the online manuscript.
