# Supplementary material for: Enteric nervous system regeneration and functional cure of experimental digestive Chagas disease with trypanocidal chemotherapy
Source: Nat Commun. 2024 May 23;15:4400. doi: 10.1038/s41467-024-48749-5 (PMC11116530; doi:10.1038/s41467-024-48749-5)
Supplement: Supplementary file 3 — Description of Additional Supplementary Files [file 41467_2024_48749_MOESM3_ESM.pdf]

Description of Additional Supplementary Files

File Name: Supplementary Data 1
Description: ***Ex vivo* bioluminescence values (fold change radiance vs uninfected controls) and post-treatment relapse calls.** This file is provided with the online manuscript.

File Name: Supplementary Data 2
Description: **Nanostring gene expression data.** The *p* values reported derive from a two-tailed *t*-test on the log-transformed normalized data for each pair of groups under comparison, assuming unequal variance. These are provided as raw p values (Columns headed “P-value”) and after adjustment using the Benjamini-Yekutieli procedure (Columns headed “BY.p.value”). This file is provided with the online manuscript.
