# Supplementary material for: Enteric nervous system regeneration and functional cure of experimental digestive Chagas disease with trypanocidal chemotherapy
Source: Nat Commun. 2024 May 23;15:4400. doi: 10.1038/s41467-024-48749-5 (PMC11116530; doi:10.1038/s41467-024-48749-5)
Supplement: Supplementary file 6 — Reporting Summary [file 41467_2024_48749_MOESM6_ESM.pdf]

nature portfolio

Corresponding author(s): Michael Lewis

Last updated by author(s): 26-01-24

Reporting Summary

Nature Portfolio wishes to improve the reproducibility of the work that we publish. This form provides structure for consistency and transparency in reporting. For further information on Nature Portfolio policies, see our Editorial Policies and the Editorial Policy Checklist.

Statistics

For all statistical analyses, confirm that the following items are present in the figure legend, table legend, main text, or Methods section.

n/a	Confirmed	
☐	☒	The exact sample size (*n*) for each experimental group/condition, given as a discrete number and unit of measurement
☐	☒	A statement on whether measurements were taken from distinct samples or whether the same sample was measured repeatedly
☐	☒	The statistical test(s) used AND whether they are one- or two-sided<br>*Only common tests should be described solely by name; describe more complex techniques in the Methods section.*
☒	☐	A description of all covariates tested
☐	☒	A description of any assumptions or corrections, such as tests of normality and adjustment for multiple comparisons
☐	☒	A full description of the statistical parameters including central tendency (e.g. means) or other basic estimates (e.g. regression coefficient) AND variation (e.g. standard deviation) or associated estimates of uncertainty (e.g. confidence intervals)
☐	☒	For null hypothesis testing, the test statistic (e.g. *F*, *t*, *r*) with confidence intervals, effect sizes, degrees of freedom and *P* value noted<br>*Give P values as exact values whenever suitable.*
☒	☐	For Bayesian analysis, information on the choice of priors and Markov chain Monte Carlo settings
☒	☐	For hierarchical and complex designs, identification of the appropriate level for tests and full reporting of outcomes
☐	☒	Estimates of effect sizes (e.g. Cohen's *d*, Pearson's *r*), indicating how they were calculated

Our web collection on statistics for biologists contains articles on many of the points above.

Software and code

Policy information about availability of computer code

Data collection	Living Image v.4.7.3., LabChartPro v.8
Data analysis	GraphPad Prism v.9, R v3.6.3, FIJI, nSolver v.4.0, Living Image v.4.7.3, LabChartPro v.8

For manuscripts utilizing custom algorithms or software that are central to the research but not yet described in published literature, software must be made available to editors and reviewers. We strongly encourage code deposition in a community repository (e.g. GitHub). See the Nature Portfolio guidelines for submitting code & software for further information.

Data

Policy information about availability of data

All manuscripts must include a data availability statement. This statement should provide the following information, where applicable:

- Accession codes, unique identifiers, or web links for publicly available datasets
- A description of any restrictions on data availability
- For clinical datasets or third party data, please ensure that the statement adheres to our policy

Source data are provided with the paper as a supplementary file.

Research involving human participants, their data, or biological material

Policy information about studies with human participants or human data. See also policy information about sex, gender (identity/presentation), and sexual orientation and race, ethnicity and racism.

Reporting on sex and gender	n/a
Reporting on race, ethnicity, or other socially relevant groupings	n/a
Population characteristics	n/a
Recruitment	n/a
Ethics oversight	n/a

Note that full information on the approval of the study protocol must also be provided in the manuscript.

Field-specific reporting

Please select the one below that is the best fit for your research. If you are not sure, read the appropriate sections before making your selection.

☒ Life sciences ☐ Behavioural & social sciences ☐ Ecological, evolutionary & environmental sciences

For a reference copy of the document with all sections, see nature.com/documents/nr-reporting-summary-flat.pdf

Life sciences study design

All studies must disclose on these points even when the disclosure is negative.

Sample size	A power calculation was performed using pilot data for total GI transit time (min) in infected vs uninfected mice ($\bar{x}1 - \bar{x}2$ = 163 – 98, mean delay = 65, SD = 35). The primary outcome effect size was ≥70% reversal of transit time delay after BZ-mediated cure of infection (target post-treatment $\bar{x}$ = 117 min). Calculations were carried out using the NC3Rs Experiment Design Assistant (https://eda.nc3rs.org.uk/) for power = 0.8 and α = 0.05. The inferred sample size was n = 10 per experiment. An additional 5 mice were allocated to the BZ treatment group to account for a predicted 2:1 ratio of cures to relapses.
Data exclusions	Sample sizes were reduced for some data sets due to attrition of mice associated with progression to a humane end-point before the end of an experiment (n=6) and imaging equipment faults (n=1).
Replication	Two independent replicates of the core in vivo experiment (treatment at 6 weeks, follow-up to 36 weeks) were conducted and the results were consistent. Only one replicate of the late treatment experiment (treatment at 24 weeks, follow-up to 48 weeks) was conducted for reasons relating to COVID pandemic-related restrictions, project duration and financial resources.
Randomization	No formal randomisation protocols were used. Age as a covariate was controlled by purchasing animals within a 2 week age range. Sex as a covariate was controlled by using only female mice. Genetics as a covariate was controlled by using an inbred mouse line and an asexual parasite clone. Weight as a covariate was controlled by weighing animals during the pre-study habituation period and swapping animals between cages in order to reduce variation in average start weights for each cage.
Blinding	Mice were allocated to groups by an investigator blinded to the groups. For downstream analysis, mice and samples were given unique alpha-numeric codes to mask the experimental conditions, but formal blinding of samples was not possible because the same investigators were involved in sample preparation i.e. necropsy and in conducting downstream assays.

Reporting for specific materials, systems and methods

We require information from authors about some types of materials, experimental systems and methods used in many studies. Here, indicate whether each material, system or method listed is relevant to your study. If you are not sure if a list item applies to your research, read the appropriate section before selecting a response.

Materials & experimental systems

n/a	Involved in the study	
☐	☒	Antibodies
☐	☒	Eukaryotic cell lines
☒	☐	Palaeontology and archaeology
☐	☒	Animals and other organisms
☒	☐	Clinical data
☒	☐	Dual use research of concern
☒	☐	Plants

Methods

n/a	Involved in the study	
☒	☐	ChIP-seq
☒	☐	Flow cytometry
☒	☐	MRI-based neuroimaging

Antibodies

Antibodies used

Primary Antibodies:
Mouse anti-HuC/D IgG clone 16A11 at 1:200, ThermoFisher, Catalog # A-21271, Lot # 2098705
Rabbit anti-nNos polyclonoal IgG at 1:500, ThermoFisher, Catalog # 61-7000, Lot # WB311534
Human anti-Hu sera ("ANNA-1") at 1:25000, a gift from Prof. Vanda Lennon, Mayo Clinic
Rabbit anti-cleaved caspase-3 (Asp175) IgG clone 269518, at 1:250, R&D Systems, Catalog # MAB835
Rat anti-GFAP monoclonal IgG clone 2.2B10 at 1:500 (1:2000 for Western Blot), ThermoFisher, Catalog # 13-0300, Lot # XD 372501, XH 352435
Rabbit anti-tubulin beta-3 (TuJ1) polyclonal IgG, at 1:500, Biolegend, Catalog # 802001, Lot # B313216

Secondary Antibodies, unless stated all from ThermoFisher and used at 1:500 dilution:
Goat anti-Mouse IgG (H+L) Highly Cross-Adsorbed Secondary Antibody, Alexa Fluor 546, Catalog # A-11030, Lot # 2026145
Goat anti-Rabbit IgG (H+L) Cross-Adsorbed Secondary Antibody, Alexa Fluor 633, Catalog # A-21070, Lot # 2079350
Goat anti-Rat IgG (H+L) Cross-Adsorbed Secondary Antibody, Alexa Fluor 546, Catalog # A-11081, Lot # 737671
Goat anti-Human IgG (H+L) Cross-Adsorbed Secondary Antibody, Alexa Fluor 647, Catalog # A-21445, Lot # 2339821
Donkey anti-Rabbit IgG (H+L) Highly Cross-Adsorbed Secondary Antibody, Alexa Fluor 488, Catalog # A-21206, Lot # 2156521
Goat Anti-Rat IgG-HRP, at 1:5000 (for western blot only), Southern Biotech, Catalog # 3030-05, Lot # J1713-M322

Validation

In-house validation: A no primary control test was performed for all antibody applications.

The anti-HuC/D mouse monoclonal antibody recognises the Elav family members HuC, HuD and Hel-N1 neuronal proteins. It does not recognize HuR, another Elav family member that is present in all proliferating cells. Application reference: Gamage et al., Neurogastroenterology and Motility 2013 and Soret et al., Gastroenterology 2020.

The rat anti-GFAP monoclonal antibody specifically binds to GFAP protein in the nervous system (both CNS and ENS). Species reactivity includes mouse amongst others. Application reference: Gordon et al., Nature Neuroscience 2021 and Chang-Jie Shi et al., Acta Pharmacologica Sinica 2023.

The rabbit anti-nNos polyclonoal antibody reacts with the ~160 kDa nNOS protein and does not exhibit any cross-reactivity with the related eNOS or iNOS proteins. Application reference: Tao et al., Neuron 2021 and McCann et al., Nature Communications 2017.

The rabbit anti-cleaved caspase-3 antibody human and mouse caspase-3 cleaved at Asp175. No cross-reactivity is reported with the full-length procaspase-3 or other caspases. Application reference: Koda et al., 2021 Nature Communications.

The rabbit anti-tubulin beta-3 (TuJ1) polyclonal antibody is well characterized and highly reactive to neuron specific Class III ß-tubulin (ßIII). TUJ1 does not identify ß-tubulin found in glial cells. TUJ1 recognizes an epitope located within the last 15 C-terminal residues. Application reference: Jongbloets et al., Nat Commununications 2017 and Radtke et al., Nat Protocols 2022.

The human anti-Hu sera has been validated in the source laboratory (Lennon lab, Mayo Clinic) where an initial adsorption of the sera with bovine liver powder was performed to remove traces of non-organ specific ANA. The testing catalog is provided here: https://www.mayocliniclabs.com/test-catalog/Overview/43431. Application reference: Lennon et al., Journal of Autoimmunity 1999 and Woods et al., American Journal of Physiology Gastrointestinal and Liver Physiology 2022.

All antibodies used in this study are commercially available (unless specified). Antibody validation has been performed by the manufacturers:
ThermoFisher (https://www.thermofisher.com/uk/en/home/life-science/antibodies/invitrogen-antibody-validation.html)
Biolegend (https://www.biolegend.com/nl-nl/%20reproducibility)
R&D Systems (https://www.rndsystems.com/products/rd-systems-approach-antibody-quality).

Additional information for each antibody is specified in the manufacturer's website which can be accessed using catalog numbers listed above.

Eukaryotic cell lines

Policy information about cell lines and Sex and Gender in Research

Cell line source(s)	Cercopithecus aethiops (African green monkey) embryonic kidney epithelial cell line MA-104 Clone 1 CRL-2378 from ATCC. The sex of the originating animal is not known.
Authentication	MA104 cells were not authenticated.
Mycoplasma contamination	Cell lines were not tested for mycoplasma contamination.
Commonly misidentified lines (See ICLAC register)	No commonly misidentified cell lines were used in the study.

Animals and other research organisms

Policy information about studies involving animals; ARRIVE guidelines recommended for reporting animal research, and Sex and Gender in Research

Laboratory animals	Mus musculus C3H/HeN and CB17 SCID mice, aged 6-8 weeks
Wild animals	No wild animals were used in the study.
Reporting on sex	The findings are based on female mice only.
Field-collected samples	No field collected samples were used in the study.
Ethics oversight	All animal procedures were performed under UK Home Office project license P9AEE04E, approved by LSHTM Animal Welfare Ethical Review Board and in accordance with Animal Scientific Procedure Act (ASPA) 1986 regulations.

Note that full information on the approval of the study protocol must also be provided in the manuscript.

Plants

Seed stocks	n/a
Novel plant genotypes	n/a
Authentication	n/a
