# Supplementary figures and images for: Enteric nervous system regeneration and functional cure of experimental digestive Chagas disease with trypanocidal chemotherapy
Source: Nat Commun. 2024 May 23;15:4400. doi: 10.1038/s41467-024-48749-5 (PMC11116530; doi:10.1038/s41467-024-48749-5)

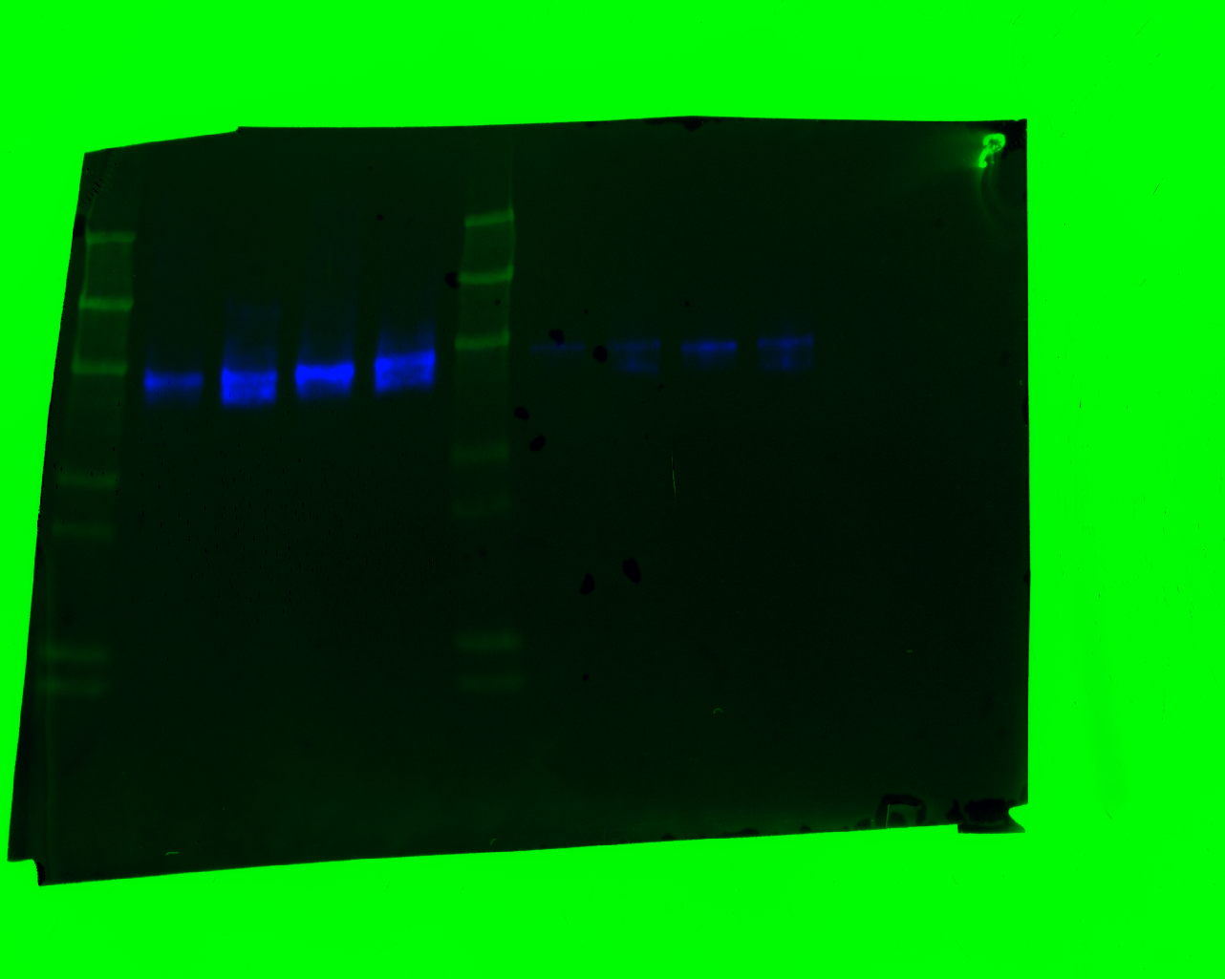

Supplement: Supplementary file 7 — Source Data [file 41467_2024_48749_MOESM7_ESM.zip › Fig 5D panel 1_anti-GFAP.tif]

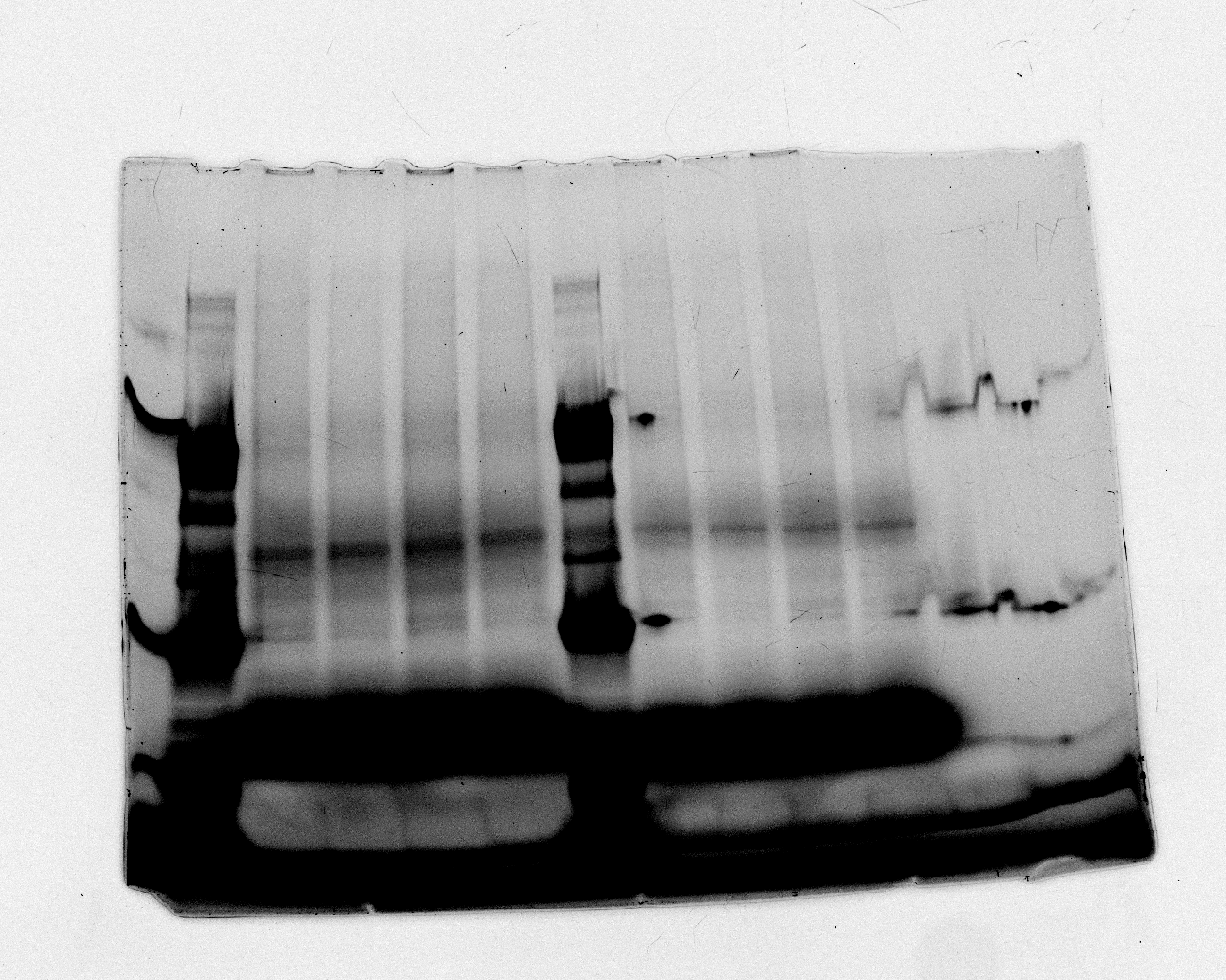

Supplement: Supplementary file 7 — Source Data [file 41467_2024_48749_MOESM7_ESM.zip › Fig 5D panel 2_total protein.tif]
